# Effects of Target Temperature Management on the Outcome of Septic Patients with Fever

**DOI:** 10.1155/2017/3906032

**Published:** 2017-11-12

**Authors:** Ye Gao, Jianjun Zhu, Chenyu Yin, Jianliang Zhu, Tao Zhu, Lijun Liu

**Affiliations:** ^1^Intensive Care Unit, Taicang Affiliated Hospital of Soochow University, Taicang 215400, China; ^2^Intensive Care Unit, The Second Affiliated Hospital of Soochow University, Suzhou 215004, China

## Abstract

**Objectives:**

To investigate the effects of target temperature management on hemodynamic changes, inflammatory and immune factors, and clinical outcomes of sepsis patients with fever.

**Methods:**

Patients diagnosed with sepsis with a core temperature of ≥39°C were randomly divided into two groups: a low-temperature group (LT group: 36.5°C–38°C) and a high-temperature group (HT group: 38.5°C–39.5°C). A target core temperature was achieved within 6 hrs posttreatment and maintained for 24 hrs. Then, the hemodynamic changes, inflammatory and immune factors, and clinical outcomes were evaluated.

**Results:**

Compared with the HT group, C-reactive protein (CRP), procalcitonin (PCT), interleukin-6 (IL-6), and tumor necrosis factor-*α* (TNF-*α*) showed a significant decrease in the LT group (*P* < 0.05). In contrast, IL-4 and IL-10 were higher in the LT group than in the HT group (*P* < 0.05). The CD4-T lymphocyte (CD4+), CD8-T lymphocyte (CD8+), and monocytic human leukocyte antigen-DR (mHLA-DR) in the LT group were higher than in the HT group (*P* < 0.05). The ICU stay and the anti-infection treatment costs were higher in the LT group (*P* < 0.05).

**Conclusion:**

Low-temperature management of patients resulted in a low level of proinflammatory cytokines. Excessive temperature control in sepsis patients with fever may be harmful.

## 1. Introduction

Sepsis remains a significant healthcare issue that may trigger high mortality and expensive healthcare cost [1]. It may progress into severe sepsis, septic shock, or even multiple organ dysfunction syndrome (MODS) if no treatment options are available immediately after diagnosis [2]. Nowadays, the mortality and prevalence of sepsis are on rise although extensive efforts have been made to better understand its pathogenesis [3].

Hyperthermia (>38°C) and hypothermia (<36°C) are the major clinical manifestations for patients with sepsis. In a previous study, Young et al. reported that elevated peak temperature in the first 24 hrs in the ICU was associated with decreased in-hospital mortality in critically ill patients with infection [4]. However, hyperthermia may contribute to tissue and organ damage, which finally results in poor prognosis. For example, Laupland et al. reported that patients with a temperature of ≥39.5°C showed a higher incidence of arrhythmia, tachycardia, severe brain injury, and even mortality compared with their counterparts with a temperature of <39.5°C [5]. Similarly, hypothermia may also induce deterioration of infection and coagulation disorder and even death as it may inhibit the migration of white blood cells and affect phagocytosis [6]. In septic rats, hypothermia may increase the prevalence of complications after infection compared with the mild hypothermia in septic rats [7]. Additionally, Yang et al. showed that controlling fever to a lower range of 36.0°C–37.5°C may induce harmful effects in patients with refractory septic shock with elevation of white blood cells and neutrophils, which implied the decreased capacity of anti-infection when compared to controlling within a higher range of 37.5°C–38.3°C [8]. In experimental sepsis rats, postconditioning hypothermia was associated with increased survival duration during experimental sepsis [9]. Moreover, in a clinical multicenter randomized controlled trial, fever control using external cooling was considered to be safe and could decrease the vasopressor requirements and early mortality in septic shock fever patients requiring vasopressors [10].

To date, there are still some disputes on temperature control for septic patients with fever. In this study, we aim to identify the temperature range that may benefit septic patients after target temperature management.

## 2. Materials and Methods

### 2.1. Patients

Septic patients with a core temperature of ≥39.0°C admitted to the ICU of the Second Affiliated Hospital of Soochow University and Taicang Affiliated Hospital of Soochow University from June 2015 to July 2016 were included in this study. Sepsis was diagnosed according to the Sepsis-3.0 diagnostic standards as previously described [11]. The inclusion criteria were as follows: (i) those diagnosed with sepsis according to the 3.0 standard, (ii) those aged 18–85 yrs, (iii) those with an expected ICU stay of at least 48 hrs, and (iv) those with a core temperature of ≥39.0°C. These patients also comprised those with septic shock. If they met the inclusion criteria, they were included immediately. Otherwise, antishock therapy should be performed according to the guidelines, and then they were included until they met the inclusion criteria. All the exclusion criteria were as follows: (i) those that received defervescence drugs (e.g., nonsteroidal anti-inflammatory drug, NSAID) and/or intravascular cooling; (ii) those with severe cardiac diseases such as obstructive myocardiopathy, myocardial infarction, and ventricular regional wall motion abnormalities; (iii) those that received agents inhibiting the inflammatory mediators (e.g., glucocorticosteroid or ulinastatin); (iv) those with refractory shock [12] as previously described; (v) those that are pregnant; (vi) those with nervous system diseases that may affect the thermoregulatory center, such as cerebral hemorrhage, craniocerebral trauma, intracranial infection, and new cerebral infarction; (vii) those that received renal replacement therapy; and (viii) those who terminated the treatment. All patients signed an informed consent form. The study protocols were approved by the Ethical Committee of the Second Affiliated Hospital of Soochow University and Taicang Affiliated Hospital of Soochow University.

### 2.2. Grouping

Patients diagnosed with sepsis with a core temperature of ≥39°C were randomly divided into two groups: a group achieving a “low-temperature” range (LT group: 36.5°C–38°C) and a group achieving a “high-temperature” range (HT group: 38.5°C–39.5°C) by physical methods including a water-flow cooling blanket and ice packs. A target core temperature was achieved within 6 hrs posttreatment and maintained for 24 hrs. All the monitoring of core temperature was performed through rectal or blood temperature methods as previously described [13].

### 2.3. Treatment

The patients were managed according to the guidelines for bundle therapy for sepsis and septic shock as previously described [14]. The major treatment options included fluid resuscitation, vasoactive agents, anti-infective therapy, and other supportive therapies such as analgesics, sedative therapy, respiratory support, blood sugar control, prevention of deep vein thrombosis, nutritional support, and stress ulcer management.

### 2.4. Data Collection

The data collected included age, sex, admission diagnosis, infection sites, ICU stay, duration and methods of temperature management, Acute Physiology and Chronic Health Evaluation II (APACHE II) score, Sequential Organ Failure Assessment (SOFA) score, drugs for analgesia and sedative therapy, way of ventilation, and temperature monitoring methods.

### 2.5. Vital Signs and Laboratory Index

The vital signs, circulating monitor indices, routine blood test, bleeding time, coagulation time, infection indices, immune inflammatory factors, blood gas analysis, and blood sugar were determined before temperature management, as well as 12 hrs and 24 hrs after temperature management.

### 2.6. Hemodynamics and the Use of Vasoactive Agent

The hemodynamics of the patients were monitored using the PICCO system as previously described [15]. The vasoactive agent used for the subjects was noradrenalin, pumped in through a central venous catheter.

### 2.7. Evaluation of Outcomes

The outcomes evaluated in the study included ICU stay, anti-infection cost, and 28-day survival rate.

### 2.8. Statistical Analysis

PASW Statistics 18 software was used for the data analysis. Measurement data that are normally distributed were presented as mean ± standard deviation, while those not normally distributed were presented as median (interquartile). Student's *t*-test or Mann–Whitney *U* test was used for intergroup comparison. Enumeration data were compared using chi-square test. R-Project software and Bionom.test were used to estimate the 95% CI of patients' proportion with a 50% decrease of the vasoactive agent. Prop.test was utilized to compare the difference. Kaplan-Meier method was used to calculate the 28-day survival. Log-rank test was used for the intergroup comparison. *P* < 0.05 was considered to be statistically significant.

## 3. Results

### 3.1. Patient Characteristics

In total, 63 patients (males: 48, females: 15, mean age: 58.54 ± 16.82 yrs) were included in this study, among whom 31 patients were assigned to the LT group and 32 patients were assigned to the HT group. No statistical differences were noticed in the sex, age, diseases, APACHE II and SOFA scores, infection source, pathogen, and complications between the two groups (*P* > 0.05, Table 1). During the 24 hrs of the temperature management, no incidence of severe cardiac arrhythmia or pressure sores was noticed in each group. Eight cases showed shivering, which was mitigated after sedative therapy.

### 3.2. Changes of Temperature and Hemodynamics at Each Time Point

**Figure 1:**
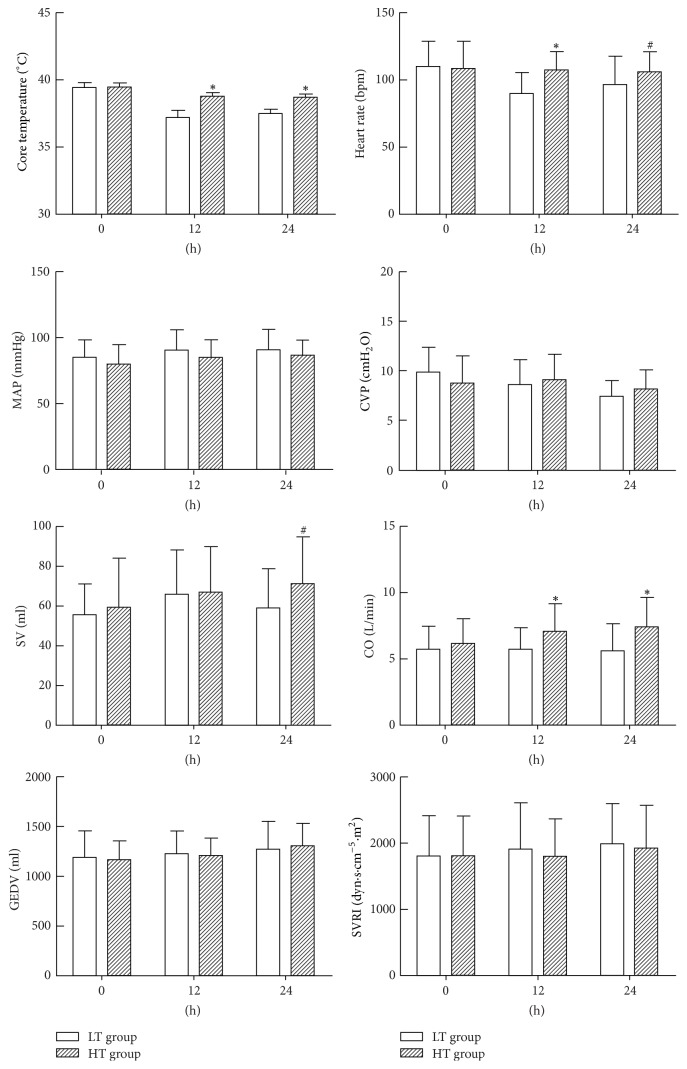
Changes of core temperature, heart rate, MAP, CVP, SV, CO, GEDV, and SVRI in different groups. ^*∗*^*P* < 0.01 versus LT group. ^#^*P* < 0.05 versus LT group. LT group: low-temperature group; HT group: high-temperature group.

Before temperature control, no statistical differences were noticed in the core temperature, heart rate, mean arterial pressure (MAP), central venous pressure (CVP), stroke volume (SV), cardiac output (CO), global end-diastolic volume (GEDV), and systemic vascular resistance index (SVRI) (*P* > 0.05, Figure 1). Within 24 hrs after temperature control, the core temperature, heart rate, SV, and CO in the LT group were statistically lower than those of the HT group (*P* < 0.05). In contrast, no statistical differences were noticed in the MAP, CVP, GEDV, and SVRI between the LT group and the HT group (*P* > 0.05).

### 3.3. Changes of Lactic Acid in Each Time Point of Temperature Control

No statistical differences were identified in lactic acid in both groups before temperature control (*P* > 0.05). About 12 hrs and 24 hrs after temperature control, the lactic acid in the LT group was significantly higher than that in the HT group (*P* < 0.05, Table 2).

### 3.4. Comparison of Vasoactive Agent Utilization

**Figure 2:**
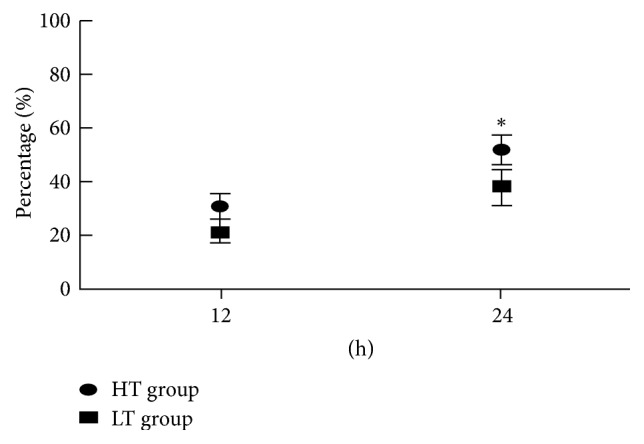
Ratio of patients with a decrease of vasoactive agent utilization of 50% compared to the baseline. ^*∗*^*P* < 0.01, comparison between LT group and HT group.

At the baseline level, 23 cases received noradrenalin in the LT group and 25 cases received noradrenalin in the HT group, which showed no statistical differences compared with the HT group (74.2% versus 78.1%, *P* = 0.714). Nevertheless, about 24 hrs after temperature control, the proportion of patients with noradrenalin decrease of 50% compared to the baseline in the HT group was statistically higher than that of the LT group (*P* < 0.01, Figure 2).

### 3.5. Comparison of Inflammatory and Immune Indices

At the baseline level, no statistical differences were noticed in the white blood cell (WBC) count, C-reactive protein (CRP), and procalcitonin (PCT) in both the low-temperature and the high-temperature groups (*P* > 0.05). In contrast, about 24 hrs after temperature control, the WBC, CRP, and PCT in the HT group were statistically higher than in the LT group (*P* < 0.05, Table 3).

No statistical differences were identified in IL-6 and TNF-*α* in both groups before the temperature control (*P* > 0.05), whereas IL-6 and TNF-*α* in the LT group showed a significant decrease compared with these of the HT group at 12 hrs and 24 hrs after temperature control (*P* < 0.05, Table 4).

IL-4 and IL-10 in both groups showed no statistical differences at the baseline levels (*P* > 0.05). Compared with the baseline level, IL-4 in the LT group showed a gradual increase at 12 hrs and 24 hrs after temperature control. Meanwhile, IL-10 reached the peak level at 12 hrs, followed by a gradual decrease at 24 hrs. In the HT group, both IL-4 and IL-10 showed a trend of decrease. Statistical differences were noticed in IL-4 and IL-10 between the two groups at 12 hrs and 24 hrs after temperature control (*P* < 0.05, Table 5).

**Figure 3:**
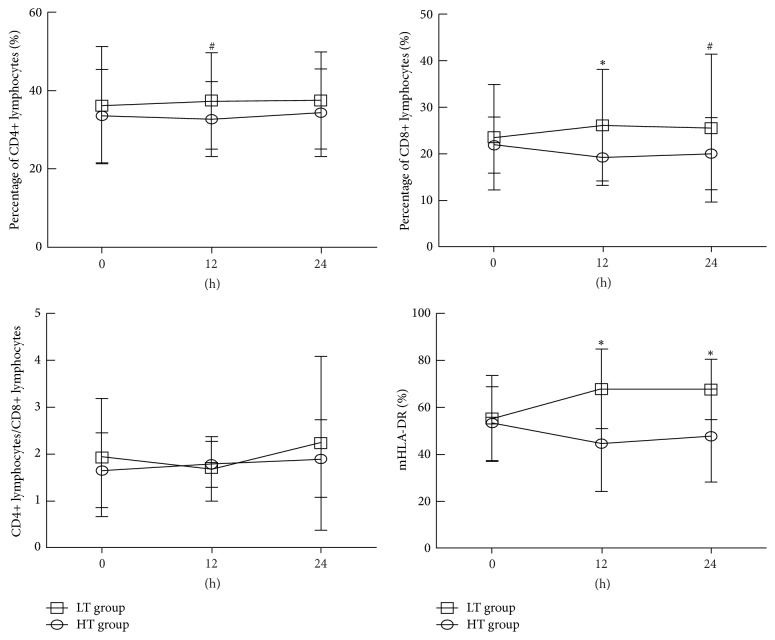
Changes of lymph node subsets (CD4+ and CD8+) and mHLA-DR antigen in LT group and HT group. ^*∗*^*P* < 0.01, comparison between HT group and LT group; ^#^*P* < 0.05, comparison between HT group and LT group.

At the baseline level, the lymphocyte subpopulation (CD4+ and CD8+) and monocytic human leukocyte antigen-DR (mHLA-DR) showed no statistical differences in both groups (*P* > 0.05). The lymphocyte subpopulation (CD4+ and CD8+) and mHLA-DR in the LT group showed a gradual increase after temperature control compared with the baseline level (*P* < 0.05). In the HT group, the lymphocyte subpopulation (CD4+ and CD8+) showed no significant difference compared to the baseline level (*P* > 0.05). For the expression of mHLA-DR, a significant increase was noticed in the LT group compared to the control group, while that of the HT group showed a significant decrease compared with the baseline level (*P* < 0.05, Figure 3).

### 3.6. Comparison of ICU Stay and Anti-Infection Therapy Cost

**Figure 4:**
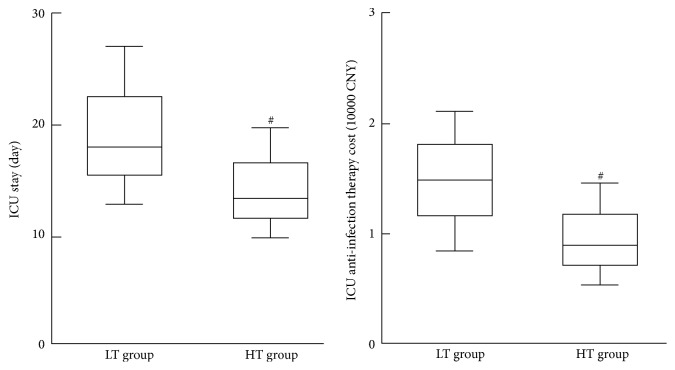
ICU hospital stay and cost of anti-infection treatment. ^#^*P* < 0.05 versus LT group.

Compared with the HT group, the ICU stay in the LT group showed a significant increase. Similarly, the anti-infection therapy cost in the LT group showed a significant increase compared with that of HT group (*P* < 0.05, Figure 4).

### 3.7. Comparison of 28-Day Survival Analysis

**Figure 5:**
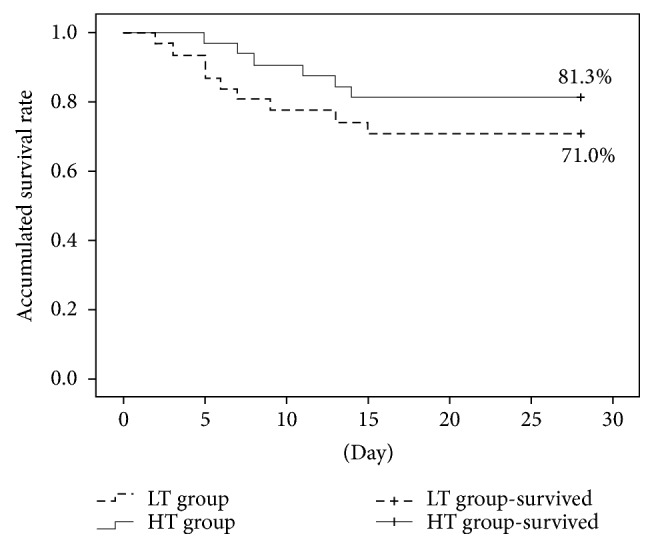
The prognosis and 28-day survival rate analysis in the two groups.

The 28-day survival rate in the LT group was 71.0%, while that in the HT group was 81.3%. Using log-rank analysis, no statistical differences were noticed in the 28-day survival rates between the two groups (*P* = 0.298, Figure 5).

## 4. Discussion

Fever is an adaptive physiological response to infection and is an important clinical sign in patients with sepsis. In the ICU, more than 90% of sepsis patients have fever [16], which is also considered an independent risk factor for death [5, 17]. As an effective strategy for organ protective therapy, body temperature management has been widely used in the treatment of fever [18]. Previous clinical studies reported that body temperature management may affect the survival of patients with sepsis [19, 20]. For instance, Peres Bota et al. [6] found that patients with septic shock combined with hypothermia showed the highest mortality. Meanwhile, patients with natural hypothermia showed a higher risk of death compared with those with fever [21]. However, there is still a lack of convincing evidence about how patients with sepsis and fever may benefit from temperature management.

An average core temperature of more than 38.3°C is defined as fever in sepsis, while a core temperature of more than 39.5°C is defined as hyperpyrexia [22]. Fever in sepsis could lead to an increased heart rate and elevation of oxygen demand, while decreased body temperature could reduce the oxygen consumption and energy requirements of the tissues. In our study, patients with hypothermia showed a decreased heart rate and SV, which then led to a decrease of cardiac output directly, thereby reducing tissue perfusion and increasing the risk of poor prognosis. Our study also showed that the levels of blood lactic acid in patients with high temperature were lower than those in patients with lower temperature after temperature management for 24 hours. This indicated that higher CO and higher oxygen delivery in patients with high temperature caused increased levels of tissue perfusion and promoted the aerobic activity and function recovery of tissues and organs, which finally contributed to the prognosis. Su et al. [23] showed that, in a sheep peritonitis sepsis model, the sheep in the fever group had higher oxygenation index, lower lactic acid level, and longer survival time compared with the sheep in other groups. The normal body temperature of sheep was in the range of 38.0°C–39.0°C. Therefore, the temperature of >39.0°C was not considered as high fever, but moderate fever. These studies suggested that patients with high temperature may have a better prognosis by means of targeted temperature management.

In addition, in order to investigate whether the dose of vasoactive agent (noradrenaline) could be reduced through target temperature management in sepsis patients with fever, the use of vasoactive agent in the two groups was analyzed in our study. Our results showed that there was no significant difference for the proportion of patients in the use of vasoactive agent in the two groups before temperature management. However, the proportion of patients in whom the dose of vasoactive agent decreased 50% baseline levels after temperature management for 24 h was significantly higher in the high-temperature group than in the low-temperature group, which may be related to higher CO and higher oxygen delivery that cause the easy correction of shock (lower level of lactic acid) in patients with high temperature. However, in a clinical multicenter randomized controlled study, the dose of vasoactive agent was reduced 2 times in septic shock patients with fever after targeted temperature management (36.5°C–37.0°C) for 48 hours, and the 14-day mortality was significantly lower [10]. The reasons for this contradictory result may be associated with temperature management time and duration and clinical heterogeneity of sepsis patients. Therefore, the relationship between the use of vasoactive drugs and the targeted temperature management in sepsis patients with fever should be further explored.

When sepsis occurs, a network is established between the endogenous inflammatory mediators including vasoactive substances, cytokines, chemokines, oxygen radicals, acute phase reactants, bioactive lipid, plasma enzyme system products, and fibrinolytic pathways. In cases of network imbalance, widespread damage of various systems and organs was induced. Proinflammatory factors such as IL-1, IL-6, and TNF-*α* could aggravate tissue injury, while anti-inflammatory factors such as IL-4, IL-10, and transforming growth factor-beta (TGF-*β*) could inhibit the inflammatory reaction. The destruction of the dynamic balance between proinflammatory factors and anti-inflammatory factors may be an important mechanism for the development of sepsis. In some experiments, fever could improve the endotoxin effects, and temperature management would affect the changes of inflammation mediators in sepsis. An animal experiment showed that, compared with the normal temperature group (36°C–38°C), the rats injected with endotoxin had reduced mortality, decreased proinflammatory factors such as IL-6 and TNF-*α*, and decreased NO in the mild-to-moderate low-temperature (34°C-35°C, 30°C-31°C) group [24]. Léon et al. [25] found that lowering body temperature could reduce the release of certain inflammatory mediators (IL-6 and TNF-*α*) and extent of oxidative response and improve the survival rate in septic rats. Huet et al. [26] revealed that hypothermia (32°C) could significantly reduce the mortality of rats with endotoxemia, which may be associated with an increased protective effect of the anti-inflammatory factor IL-10. In this study, a decrease of WBC, CRP, PCT, and proinflammatory factors (IL-6 and TNF-*α*) and an increase of anti-inflammatory factors (IL-4 and IL-10) were noticed in the low-temperature group after 24 hours of temperature management, while these indexes had little changes in the high-temperature group after 24 hours of temperature management. This indicated proper reduction of body temperature to 36.0°C–38.5°C; the endotoxin induced body excessive inflammatory reaction was inhibited, and the survival rate of patients increased. For these reasons, the reduction of body temperature may be related to the decrease of metabolism and energy demands of inflammatory cells, the reduction of proinflammatory cytokines (IL-6 and TNF-*α*) release, and the increase of the anti-inflammatory cytokines (IL-4 and IL-10) contents.

In addition, our study also observed that there were no significant differences in the levels of lymphocytes subpopulations (CD4+ and CD8+) and mHLA-DR at the beginning of temperature management in the two groups. The levels of lymphocytes subpopulations (CD4+ and CD8+) and mHLA-DR were increased in the low-temperature group after 24 hours of temperature management. No significant differences were noticed in the levels of CD4+ and CD8+ in the high-temperature group after 24 hours of temperature management, whereas mHLA-DR showed a decreased trend. Lymphocytes are the main cells of the adaptive immune response, which are involved in the innate immune response. They play a vital role in the immune response against pathogenic microorganisms. There is a serious lack of important immune cells such as CD4+ T-cells, CD8+ T-cells, B-cells, dendritic cells, and mononuclear cells in sepsis. HLA-DR is a class MHC-II molecule in the immune index and plays an important role in the monocyte antigen presenting process. The expression level of mHLA-DR is the most commonly used biomarker in the evaluation of the immune status of sepsis, and its persistent low expression is the main feature of immunosuppression stage in sepsis patients [27]. Some studies found that mHLA-DR could be used to assess the prognosis, and its persistent low expression (the positive rate was below 20%–40%) is also an independent risk factor for septic shock and the incidence of nosocomial infection in ICU hospitalization patients [28, 29]. However, the lymphocyte subsets (CD4+ and CD8+) and mHLA-DR only reflect part of the body's immune function. The changes of immune status in patients with sepsis are very complex and dynamic, and the heterogeneity among patients is great; therefore, the use of these indicators for the assessment of the overall immune status needs to be discussed.

For the effects of temperature management on the outcome of septic patients, we investigated the ICU stay and anti-infection therapy cost. After achieving a target temperature, the ICU stay in the high-temperature group was comparatively lower than that in the low-temperature group, together with the anti-infection therapy cost for the ICU hospitalization. Meanwhile, the 28-day survival rate in the high-temperature group was higher than that in the low-temperature group (81.3% versus 71.0%). Although no statistical differences were identified (*P* = 0.298), this indicated that septic patients in the high-temperature group showed better outcome and prognosis than those in the low-temperature group.

There are really some limitations in this study. Septic patients were included in separate ICUs from two different hospitals, which may trigger some differences for the procedures except for temperature management. No subgroup analysis was carried out in this study as the sample size was not large enough, which may affect the outcome to some extent. For the utilization of vasoactive agents, noradrenalin is the major agonist for the *α* receptor, but it may affect the cardiac *β*1 receptor. However, in this study, we do not analyze the effects of such agent on the heart rate and CO, which may cause differences in the results. For the analysis of 28-day survival rate, no statistical differences were noticed between the two groups which may be related to the small sample size, duration of temperature management, and disease severity.

## 5. Conclusions

Low temperature results in a low level of proinflammatory cytokines in septic patients with fever, and the quantity of lymphocyte subsets becomes high. Meanwhile, the ICU stay in the high-temperature group was comparatively lower than that in the low-temperature group, together with the anti-infection therapy cost for the hospitalization. Excessive temperature control may be harmful to septic patients.

## Figures and Tables

**Table 1:** Clinical characteristics at baseline.

Variable	Low-temperature group (*n* = 31)	High-temperature group (*n* = 32)	*T* or *χ*^2^ or Mann–Whitney *U* value	*P* value
Age (yr)	56.90 ± 14.75	60.13 ± 18.70	0.758	0.452
Gender (male/female)	23/8	25/7	0.134	0.714
Complication (cases)			1.417	0.702
Hypertension	12	9		
Diabetes	5	7		
COPD	4	3		
Old cerebral infarction	7	10		
APACHE II score	22 (20~30)	24 (21~31)	399.00	0.538
SOFA score	9 (7~13)	9 (6~13)	491.50	0.950
Classification of diseases (cases)			0.176	0.674
Medical diseases	20	18		
Surgical diseases	11	14		
Infective site			2.816	0.412
Lung	19	24		
Abdominal cavity	9	7		
Urinary passage	1	1		
Catheter relevance	2	0		
Infectious pathogens			0.718	0.949
Gram-positive bacteria	5	6		
Gram-negative bacteria	15	13		
Fungal infection	2	2		
Mixed infection	6	6		
Others	3	5		
Number of mechanical ventilation instances (rate)	11 (35.5%)	13 (40.6%)	1.417	0.702
Accuracy of anti-infective therapy			1.171	0.557
Yes	24	25		
No	4	2		
Uncertainty	3	5		

APACHE II score: Acute Physiology and Chronic Health Evaluation II score; SOFA score: Sequential Organ Failure Assessment score.

**Table 2:** Levels of lactic acid (mmol/L) at different time points in the two groups.

Time (h)	LT group (*n* = 31)	HT group (*n* = 32)	Mann–Whitney *U* value	*P* value
0	1.20 (1.10~1.60)	1.15 (0.83~2.30)	495.00	0.989
12	1.75 (1.20~2.43)	1.20 (0.70~1.95)	349.50	***0.043***
24	1.80 (1.18~2.50)	1.05 (0.90~1.90)	341.50	***0.033***

**Table 3:** Changes of WBC, CRP, and PCT at different time points of temperature management.

	Time (h)	LT group (*n* = 31)	HT group (*n* = 32)	Mann–Whitney *U* value	*P* value
WBC (10^9^/L)	0	15.70 (12.30~26.52)	17.25 (10.80~27.37)	479.00	0.983
	24	10.57 (6.39~14.10)	13.90 (9.60~23.08)	331.00	***0.023***

CRP (mg/L)	0	161.51 (81.15~276.51)	164.60 (85.68~205.71)	467.00	0.855
	24	89.92 (43.08~144.03)	137.95 (76.05~181.34)	327.00	***0.031***

PCT (*μ*g/L)	0	4.73 (2.07~9.46)	5.42 (1.69~7.51)	473.50	0.757
	24	2.65 (0.70~5.65)	5.38 (2.36~12.11)	341.50	***0.034***

**Table 4:** Changes of proinflammatory factors at different time points of temperature management.

	Time (h)	LT group (*n* = 31)	HT group (*n* = 32)	Mann–Whitney *U* value	*P* value
IL-6 (pg/ml)	0	40.40 (13.68~105.01)	37.19 (15.16~108.76)	491.00	0.945
	12	20.19 (17.34~49.04)	43.57 (21.33~114.65)	361.00	0.045
	24	17.54 (13.11~98.01)	38.40 (29.08~161.54)	182.00	0.010

	Time (h)	LT group (*n* = 31)	HT group (*n* = 32)	*T* value	*P* value

TNF-*α* (pg/ml)	0	2.45 ± 2.33	2.20 ± 1.96	0.450	0.654
	12	1.56 ± 0.65	2.11 ± 0.80	2.950	0.005
	24	1.66 ± 0.96	2.46 ± 1.26	2.837	0.006

**Table 5:** Changes of anti-inflammatory factors at different time points of temperature management.

	Time (h)	LT group (*n* = 31)	HT group (*n* = 32)	*T* value	*P* value
IL-4 (pg/ml)	0	3.64 ± 0.64	3.63 ± 0.62	0.044	0.965
	12	4.56 ± 1.27	3.56 ± 0.80	3.753	0.000
	24	4.90 ± 0.95	3.46 ± 0.65	3.906	0.000

	Time (h)	LT group (*n* = 31)	HT group (*n* = 32)	Mann–Whitney *U* value	*P* value

IL-10 (pg/ml)	0	8.91 (6.14~45.94)	7.75 (5.32~21.75)	449.00	0.305
	12	10.03 (5.91~25.34)	6.96 (4.45~8.91)	325.50	0.009
	24	8.80 (5.47~15.82)	5.54 (3.98~6.83)	292.00	0.047
